# Supplementary material for: Real-time measurement of spatial distance to external breakage hazards of transmission pole tower based on monocular vision
Source: PLoS One. 2025 Jul 11;20(7):e0326254. doi: 10.1371/journal.pone.0326254 (PMC12250280; doi:10.1371/journal.pone.0326254)
Supplement: S1 File — (DOCX) [file pone.0326254.s001.docx]

**Table 1 Original data set of Figure 8**

| Epoch | (a) | | (b) | |
| --- | --- | --- | --- | --- |
|  | Unoptimised | Transformer | Unoptimised | Transformer |
| 0 | 4.75 | 4.75 | 0.04 | 0.00 |
| 5 | 2.85 | 2.43 | 0.47 | 0.25 |
| 10 | 2.82 | 2.05 | 0.58 | 0.26 |
| 15 | 2.76 | 1.82 | 0.59 | 0.28 |
| 20 | 2.67 | 1.78 | 0.60 | 0.30 |
| 25 | 2.58 | 1.73 | 0.61 | 0.31 |
| 30 | 2.54 | 1.67 | 0.62 | 0.31 |
| 35 | 2.50 | 1.64 | 0.63 | 0.32 |
| 40 | 2.48 | 1.63 | 0.63 | 0.32 |
| 45 | 2.46 | 1.62 | 0.65 | 0.33 |
| 50 | 2.43 | 1.62 | 0.65 | 0.35 |

**Table 2 Original data set of Figure 9**

| Method | IoU_3D_=0.7 | | | IoU_BEV_=0.7 | | |
| --- | --- | --- | --- | --- | --- | --- |
|  | Easy | Medium | Hard | Easy | Medium | Hard |
| F-PoinNet | 38.23 | 24.45 | 19.68 | 49.65 | 27.88 | 25.67 |
| F-PoinNet+Transformer | 45.27 | 26.66 | 24.24 | 56.23 | 43.89 | 29.85 |
| PVRCNN | 41.87 | 25.03 | 23.10 | 53.20 | 29.35 | 26.54 |
| PVRCNN+Transformer | 45.88 | 27.06 | 24.56 | 58.25 | 35.67 | 31.08 |

**Table 3 Original data set of Figure 11**

| Times | (a) | | | | | (b) | | | | | (c) | | | | |
| --- | --- | --- | --- | --- | --- | --- | --- | --- | --- | --- | --- | --- | --- | --- | --- |
|  | DRM | Did-m3d | FAS | SGM3D | This paper | DRM | Did-m3d | FAS | SGM3D | This paper | DRM | Did-m3d | FAS | SGM3D | This paper |
| 1 | 74.9 | 72.7 | 78.5 | 68.9 | 81.8 | 57.2 | 60.0 | 62.6 | 55.2 | 64.2 | 54.5 | 54.9 | 55.0 | 54.1 | 55.9 |
| 3 | 75.4 | 72.1 | 81.2 | 70.3 | 81.9 | 573. | 60.4 | 62.1 | 55.2 | 64.1 | 54.5 | 54.8 | 55.1 | 54.4 | 55.8 |
| 5 | 75.3 | 72.5 | 78.9 | 70.3 | 81.7 | 57.2 | 60.5 | 62.3 | 55.3 | 64.2 | 54.6 | 54.8 | 55.1 | 54.3 | 55.9 |
| 7 | 75.2 | 72.1 | 79.6 | 70.4 | 81.8 | 57.2 | 60.2 | 62.3 | 55.1 | 64.2 | 54.5 | 54.8 | 55.1 | 54.4 | 55.9 |
| 9 | 75.0 | 72.1 | 80.2 | 70.5 | 82.0 | 57.3 | 60.8 | 62.2 | 55.2 | 64.2 | 54.5 | 54.7 | 55.1 | 54.4 | 55.9 |

**Table 4 Original data set of Figure 12**

| Times | (a) | | | | | (b) | | | | | (c) | | | | |
| --- | --- | --- | --- | --- | --- | --- | --- | --- | --- | --- | --- | --- | --- | --- | --- |
|  | DRM | Did-m3d | FAS | SGM3D | This paper | DRM | Did-m3d | FAS | SGM3D | This paper | DRM | Did-m3d | FAS | SGM3D | This paper |
| 1 | 71.9 | 75.1 | 77.6 | 84.8 | 91.1 | 62.3 | 62.5 | 66.8 | 72.1 | 76.2 | 49.8 | 51.9 | 54.0 | 56.7 | 57.2 |
| 3 | 71.8 | 75.2 | 78.4 | 84.8 | 91.1 | 62.5 | 62.7 | 66.9 | 72.5 | 76.5 | 49.9 | 51.8 | 54.1 | 56.7 | 57.7 |
| 5 | 72.0 | 75.2 | 78.4 | 85.0 | 91.0 | 62.5 | 62.7 | 67.5 | 72.5 | 76.2 | 50.0 | 52.0 | 54.4 | 56.8 | 57.0 |
| 7 | 73.4 | 75.4 | 79.8 | 84.9 | 90.7 | 62,0 | 62.7 | 66.4 | 72.5 | 76.2 | 50.0 | 51.9 | 54.3 | 56.0 | 57.7 |
| 9 | 72.5 | 75.5 | 81.4 | 85.0 | 90.7 | 61.8 | 62.6 | 65.9 | 72.3 | 75.6 | 50.0 | 51.9 | 54.4 | 56.5 | 57.7 |

**Table 5 Original data set of Figure 13**

| Experimental data | (a) | | | (b) | | |
| --- | --- | --- | --- | --- | --- | --- |
|  | Real | Test | Accuracy | Real | Test | Accuracy |
| 1 | 35.4×10^4^ | 33.7×10^4^ | 95.30% | 9.1×10^4^ | 8.4×10^4^ | 91.95% |
| 2 | 8.7×10^4^ | 8.5×10^4^ | 97.81% | 3.0×10^4^ | 2.9×10^4^ | 95.83% |
| 3 | 19.8×10^4^ | 19.1×10^4^ | 96.57% | 6.5×10^4^ | 6.1×10^4^ | 93.90% |
