# Supplementary material for: Real-time measurement of spatial distance to external breakage hazards of transmission pole tower based on monocular vision
Source: PLoS One. 2025 Jul 11;20(7):e0326254. doi: 10.1371/journal.pone.0326254 (PMC12250280; doi:10.1371/journal.pone.0326254)
Supplement: Attachment — Submitted filename: Response to reviewers comments.doc [file pone.0326254.s003.doc]

Review Comments to the Author

Please use the space provided to explain your answers to the questions above. You may also include additional comments for the author, including concerns about dual publication, research ethics, or publication ethics. (Please upload your review as an attachment if it exceeds 20,000 characters)

Reviewer #1:

This paper proposes a pseudo-point cloud distribution optimization method based on Transformer networks, which demonstrates excellent performance in improving target point cloud features and enhancing the efficiency of 3D object detection. Additionally, it introduces technological innovations such as the Geometric Constraint Depth Refinement (GCDR) module, further boosting measurement accuracy and stability.

However, I have some suggestions, as follows.

1) The article merely elaborates on the importance of safety monitoring for transmission towers and lacks a detailed comparison and analysis of existing technologies for predicting external damage risks to transmission towers. The authors are advised to include a comprehensive review of existing technologies in the introduction and clearly identify the shortcomings of current technologies.

Reply: Thank you for the suggestions. The review of existing technologies and detailed comparison of existing technologies for predicting external damage risks to transmission towers have been explained in introduction section. As follows:

**A complementary literature review:**

M. M. Hosseini et al. addressed the inefficient and time-consuming problem of manual operations in the process of power tower damage assessment by proposing an automated model that used an unmanned aerial vehicle (UAV) to capture images and transmit them in real time to an intelligent damage classification and assessment unit. The model's integration of four convolutional neural networks facilitated the learning of the tower condition from images, the extraction of image features, and the training of automated intelligent tools to replace manual fault location and damage assessment [19]. Chen et al. proposed a comprehensive risk assessment framework based on transmission tower geometry and topography to address the problem that transmission towers are prone to collapsing and triggering large-scale power outages and electrocution risks when subjected to heavy rainfall and flooding. A series of new point cloud segmentation and fitting algorithms are developed to accurately estimate the tilt angle of transmission towers by utilizing 3D point cloud data acquired from aerial LiDAR [20].

[19]	M. M. Hosseini, A. Umunnakwe, M. Parvania, and T. Tasdizen. "Intelligent damage classification and estimation in power distribution poles using unmanned aerial vehicles and convolutional neural networks." IEEE Trans. Smart Grid, vol. 11, no. 4, pp. 3325-3333, January, 2020, DOI: 10.1109/TSG.2020.2970156.

[20]	M. Chen, T. O. Chan, X. Wang, M. Luo, Y. Lin, H. Huang, Y. Huang. "A risk analysis framework for transmission towers under potential pluvial flood-LiDAR survey and geometric modelling," Int J Disast Risk Re, vol. 50, pp. 101862, November, 2020, DOI: 10.1016/j.ijdrr.2020.101862.

**Detailed analysis of current technology deficiencies:**

Combined with the above, it can be observed that scholars at home and abroad have conducted various researches on PPC distribution optimization, and have achieved good research results in many areas. However, there is a PPC long-tailed distribution effect in the process of point cloud image acquisition using LiDAR technology for transmission tower external damage risk detection. In the existing transmission tower external damage risk prediction technology, the processing time of the traditional LiDAR technology is about 3-5s, and the data processing and analysis is too time-consuming to meet the real-time demand. In addition, the completeness of the feature extraction of the towers is only about 97%, and there is a 3% error between the distribution of PPC at the edges of the towers and the distribution of the internal area to be determined, which limits the accuracy of the prediction model. And there are still some research gaps on the limitations of stereo vision in long-distance measurement.

2) The submission contains some formulas and charts that lack sufficient explanation and description. For instance, Figures 4 and 6 lack necessary textual explanations and annotations are not detailed enough. The authors should provide more detailed annotations and explanations for these formulas and charts, including more detailed labeling of specific steps and data flow between modules. Formulas (2), (3), and (4) lack specific explanations and should be briefly explained before or after the formulas to make the mathematical equations easier to understand.

Reply: Thank you very much for reviewing the manuscript. Necessary textual explanations of Figures 4 and 6 and Equations (2), (3), and (4) have been added. The details are given below:

**Figure 4:**

Figure 4 illustrates the structure of CenterMask network, which consists of spatial attention module (SAM) and feature channel attention module (FCAM). First, the input image is extracted from the feature image by the backbone network and fed into the feature pyramid network (FPN) to obtain the multi-scale feature image. Then, the image features are localized by the predictive bounding box module of the full convolutional single-stage target detection (FCOS). Finally, the image segmentation is completed by the spatial attention-guided mask (SAG-Mask). This process not only effectively extracts the target features in the image, but also enhances the ability of the model to recognize the target region through the attention mechanism.

**Figure 6:**

Figure 6 illustrates the complete process of the real-time measurement method for the spatial distance to external break hazards of transmission towers based on monocular vision. The method commences with the acquisition of the PCD of the transmission lines, subsequently followed by a series of pre-processing steps that include noise reduction and contrast enhancement. Concurrently, the optimized depth of the foreground targets is integrated with the original images to generate the PPCD.

**Equation (2):**

After the attention weighting process, the PC FV expression formula $S_{a}$ is shown in Equation (2).

$S_{a}=soft\max(\frac{qk^{T}}{\sqrt{d_{i}}})v$ (2)

In Equation (2), $soft\max$ denotes the Softmax function, it is mainly used to normalize the attention scores, ensuring that the weights of each FV sum to 1.

**Equation (3):**

Equation (3) is used to compute the weighted FVs after the self-attention layer processing, which is used to further optimize the global consistency of the point cloud features.

**Equation (4):**

The final output of Transformer's FV expression formula is shown in Equation (4).

$f_{enh}=LayerNorm(f_{attn}+FeedForward(f_{attn}))$ (4)

Equation (4), $f_{enh}$ denotes the FV of the final output of Transformer. $FeedForward$ denotes the feed-forward network of Transformer network.

3) Although there is some technical description of the pseudo-point cloud distribution optimization method based on Transformer networks and the depth optimization method based on 2D instance segmentation and geometric constraints, not much detailed description is provided. The authors are advised to add more technical details, including specific steps of the algorithm, parameter settings, and experimental environments.

Reply: Thank you for your comments. The experimental setup and parameters have been explained and more specific steps of the algorithm have been added. As follows:

**The specific steps of the algorithm is shown as follows:**

The decoder module of the transformer facilitates the restoration of the FV output by the encoder to the spatial distribution of the PC. The features are then fused at multiple scales through the use of MHSA, which optimizes the detailed information of the PC distribution. Ultimately, the error between the optimized PPC and the actual PC is calculated by the chamfer distance (CD) loss function, and the network parameters are adjusted by back-propagation.

**The parameter settings is shown as follows:**

The model's PointNet++ uses two Set Abstraction layers, and each layer contains 512 points. Transformer: Six Transformer blocks are used, and each block contains eight attention heads. Convolution layer: 4 convolution layers are used, and each convolution layer has 32 3x3 filters. Fully connected layer: Two fully connected layers are used. Moreover, the first fully connected layer has 128 neurons and the second fully connected layer has 64 neurons.

**The and experimental environments is shown as follows:**

The experimental hardware environment is GeForce RTX 3090 GPU, and the memory size is 48 GB. The CPU is Intel Core i9 and the RAM is 512GB. The operating system is Ubuntu 20.04 LTS and the programming language is Python 3.8.

4) The experimental results presented in the article lack sufficient explanation and analysis of the experimental data. For example, for experimental results in different difficulty scenarios, the authors simply list accuracy, recall, and F1 scores without in-depth discussion of the experimental results. The authors should increase the detailed explanation and analysis of the experimental data, including the source of the experimental data, a detailed description of the experimental process, and an assessment of the reliability and validity of the experimental results.

Reply: Thank you for your advice. Interpretation and analysis of experimental data and results have been supplemented in the manuscript. As follows:

**Detailed interpretation and analysis of experimental data:**

It can be inferred that as the complexity of the scene increases, the efficacy of the proposed model diminishes. This phenomenon may be attributed to the fact that the target object in the simple scene is more discernible and the background interference is minimal, thereby enabling the model to extract the target features with greater precision. However, in moderate and difficult scenes, the target object has severe occlusion, illumination change, or complex background, which poses a great challenge to the model in feature extraction and depth estimation.

**The results for accuracy, recall and F1 scores are discussed and analyzed:**

**Table 3. Comparison of the testing result of different methods**

| Method | Precision (%) | | | Recall (%) | | | F1score (%) | | | Average FPR (%) |
| --- | --- | --- | --- | --- | --- | --- | --- | --- | --- | --- |
|  | Easy | Medium | Hard | Easy | Medium | Hard | Easy | Medium | Hard |  |
| DRM | 85.02 | 75.58 | 60.24 | 82.38 | 70.48 | 55.57 | 83.68 | 72.94 | 57.81 | 15.00 |
| Did-m3d | 88.97 | 77.65 | 64.32 | 85.56 | 73.24 | 59.98 | 87.23 | 75.38 | 62.07 | 12.34 |
| FAS | 90.01 | 80.27 | 68.88 | 86.78 | 76.56 | 65.90 | 88.37 | 78.37 | 67.36 | 10.21 |
| SGM3D | 87.80 | 79.85 | 66.34 | 86.36 | 76.25 | 62.30 | 87.07 | 78.01 | 64.26 | 14.56 |
| This study | 91.15 | 83.26 | 70.97 | 89.55 | 81.98 | 69.04 | 90.34 | 82.62 | 69.99 | 8.90 |

In Table 3, the accuracy, recall, and F1 score of the proposed method are superior to other methods in different difficulty scenes. Moreover, the detection performance is obviously superior in simple and medium difficulty scenes. This shows that this method has high accuracy and robustness in dealing with 3D target detection tasks of varying difficulty. Comparing the FPR of the five methods, the FPR of DRM is the highest at 15%. This indicates that there is a 15% chance that the DRM method will incorrectly predict all negative samples as positive samples. This may indicate that the DRM method faces challenges in accurately distinguishing between positive and negative samples, especially in complex or challenging scenarios. The DRM method relies primarily on a deep regression model and is sensitive to occlusion, illumination changes, and background clutter in complex scenes. In medium and high complexity scenes, the target object may be partially occluded or appear close to the background depth. This can hinder the model's ability to accurately distinguish the target from the background, thereby reducing the detection accuracy. The feature fusion method of DID-M3D is simple, and it can't effectively deal with the deep overlap between the backgrounds of the target area. The FAS method ignores the comprehensive consideration of multi-scale features and local features when dealing with complex scenes. It is difficult to effectively deal with local occlusion and background interference of targets. The FPR of the proposed method is 8.90%, which is the lowest among all methods, indicating that it has the best performance in controlling false positives.

Therefore, the authors are advised to make major revisions before resubmitting.

Reviewer #2:

The authors presented a real-time spatial distance measurement approach using monocular vision together with a Transformer network, enabling the prediction of external breakage hazards in transmission towers. They demonstrated the effectiveness of their method on the KITTI dataset as well as actual tower data, and compared it with other methods.

Strengths:

Their method is well-explained, and the use of monocular vision and Transformer networks contributes to the power engineering. The experiments, figures and tables are clear and support their findings.

Weakness:

1. The authors only compared different method on the KITTI dataset, it would be important to use different methods on the actual tower data too.

Reply: Thank you for your comments. Different methods (such as DRM, Did-m3d, FAS, SGM3D) on the actual tower data have been used to compare with the proposed method in this study. Please see details as follows:

The comparison results of DRM, Did-m3d, FAS, SGM3D and the methods proposed in this study are shown in Table 4.

**Table 4 Comparison of actual data testing by different methods**

| Method | Accuracy (%) | | | Time overhead (s/ frame) | |
| --- | --- | --- | --- | --- | --- |
|  | Data set 1 | Data set 2 | Data set 3 | Data preprocessing | Model reasoning |
| DRM | 90.21 | 92.17 | 93.55 | 2.15 | 1.47 |
| Did-m3d | 91.53 | 93.01 | 94.05 | 1.23 | 0.94 |
| FAS | 92.00 | 93.54 | 94.52 | 0.91 | 0.76 |
| SGM3D | 91.87 | 93.22 | 94.26 | 1.34 | 1.02 |
| This study | 93.63 | 96.82 | 95.24 | 0.48 | 0.32 |

In Table 4, the accuracy of the proposed method is higher than other methods in all datasets, with an average accuracy of 95.23%. The preprocessing time of the proposed method is 0.48 s/frame, which is the lowest among all the methods. This shows that this method is more efficient in the data preprocessing stage. This may be because the processing flow of PC data is optimized. Meanwhile, it also shows that the proposed method can extract and analyze the PCD of transmission towers more efficiently in practical application.

2. Since this is a “real-time” measurement method, maybe I missed it, I am wondering how much time it will take to run a trained model on the test data. It would be also interesting to compare the running time for different methods.

Reply: Thank you for your comments. The running time for different methods has been compared and analyzed. Please see details in Table 4 as follows:

In Table 4, the accuracy of the proposed method is higher than other methods in all datasets, with an average accuracy of 95.23%. The preprocessing time of the proposed method is 0.48 s/frame, which is the lowest among all the methods. This shows that this method is more efficient in the data preprocessing stage. This may be because the processing flow of PC data is optimized. Meanwhile, it also shows that the proposed method can extract and analyze the PCD of transmission towers more efficiently in practical application.

Language and Formatting:

There are many grammatical errors in the manuscript. The authors should proofread it very carefully. For example, in the Abstract, “The innovation of the study lied in the optimization of the pseudo point cloud distribution using the Transformer network” should be corrected, “ … lied in …”. In the Conclusion, “This result indicated that optimizing the depth information of foreground targets couldsignificantly improve the accuracy of foreground targets in PPC” also requires update.

Reply: Thank you for the suggestions. The language has been revised. For example:

The sentence “The innovation of the study lied in the optimization of the pseudo point cloud distribution using the Transformer network” was changed to “The innovation of the study was the optimization of the pseudo point cloud distribution using the transformer network, which effectively captured the global dependencies and improved the global consistency and local detail accuracy of the pseudo point clouds.”

The sentence “This result indicated that optimizing the depth information of foreground targets couldsignificantly improve the accuracy of foreground targets in PPC” was changed to “This result suggested that by optimizing the depth information of foreground targets, the accuracy of foreground targets in PPC could be improved, thus increasing the overall detection efficiency.”
