# Supplementary material for: Real-time measurement of spatial distance to external breakage hazards of transmission pole tower based on monocular vision
Source: PLoS One. 2025 Jul 11;20(7):e0326254. doi: 10.1371/journal.pone.0326254 (PMC12250280; doi:10.1371/journal.pone.0326254)
Supplement: Attachment — Submitted filename: Response to reviewers comments.docx [file pone.0326254.s004.docx]

Journal Requirements:

Please review your reference list to ensure that it is complete and correct. If you have cited papers that have been retracted, please include the rationale for doing so in the manuscript text, or remove these references and replace them with relevant current references. Any changes to the reference list should be mentioned in the rebuttal letter that accompanies your revised manuscript. If you need to cite a retracted article, indicate the article’s retracted status in the References list and also include a citation and full reference for the retraction notice.

Reply: Thank you very much for your comments. The reference list is complete and correct, and we do not have cited papers that have been retracted.

Additional Editor Comments:

The reviewers have acknowledged the improvements. However, they still have some minor concerns which need to be addressed so it is suggested to address point-wise all the remaining concerns. Please make improvements or modifications in the manuscript where indicated or is required as per the reviewers' comments.

Reply: Thank you very much for your comments. We have carefully revised the paper according to the reviewer’s comments. We believe that after revision, it can meet the requirements of the reviewers.

Review Comments to the Author

Please use the space provided to explain your answers to the questions above. You may also include additional comments for the author, including concerns about dual publication, research ethics, or publication ethics. (Please upload your review as an attachment if it exceeds 20,000 characters)

Reviewer #1:

This paper proposes an optimization for pseudo point cloud (PPC) power distribution using transformer-based approaches. Building upon this foundation, it integrates lidar-based Simultaneous Localization and Mapping (SLAM) with deep learning to enhance the collaborative robotic surveying accuracy. The experimental design is well-conceived, and the conclusions offer valuable reference significance for intelligent inspection of power transmission lines and the development of visual technologies in electrical engineering. After review, I consider the paper demonstrates clear logical structure and coherent reasoning that meets publication standards. However, the following issues require further refinement before final acceptance:

Revision Suggestions:

1) While the technical details of the proposed transformer-based pseudo point cloud (PPC) distribution optimization method and the depth refinement approach integrating 2D instance segmentation with geometric constraints are well-improved, the authors are advised to enhance readability by adding intuitive explanations or citing relevant literature following this section.

Reply: Thank you very much for the suggestions. More details on PPC allocation optimisation with 2D instance splitting have been added. And relevant literature has been cited. As follows:

PointNet++, on the other hand, by introducing local feature capture, multi-scale feature fusion and hierarchical architecture, is able to improve its ability to handle complex PCDs, enabling it to perform well in a variety of PC-related tasks [25].

The 2D target detector can effectively detect foreground targets in images with complex background situations and can clearly distinguish between foreground and background. The core idea of this stage is to extract the target region in the image by 2D target detection and estimate its depth using deep learning models [31]. Compared with the traditional method, the 2D instance segmentation method can deal with the target boundary more accurately, thus improving the depth optimisation of the foreground target.

For long-range targets, the GCDR module is able to optimise the depth estimation of the target and improve the depth accuracy of long-range targets. It is assumed that the 3D height prediction of each object is generated by adding a 3D height regression head to the CentreMask model, and the 3D height has Laplace distribution randomness[34]. By combining the 3D height prediction of the target and the depth regression head, the randomness of the Laplace distribution is exploited for the correction of depth estimation.

[25]	Y. Chen, Z. Li, Q. Li, and M. Zhang, "Pose estimation algorithm based on point pair features using PointNet++," Complex. Intell. Syst., vol.10, no. 5, pp. 6581-6595, Oct. 2024, 10.1007/s40747-024-01508-x.

[31]	X. Li, H. Bagher-Ebadian, S. Gardner, J. Kim, M. Elshaikh, B. Movsas, D. Zhu, and I. J. Chetty, "An uncertainty-aware deep learning architecture with outlier mitigation for prostate gland segmentation in radiotherapy treatment planning," Med. Phys., vol. 50, no. 1, pp. 311-322, Jan. 2023, 10.1002/mp.15982.

[34]	C. Ge, R. Zhang, Y. Jiang, B. Li, and Y. He, "A 3-D dynamic non-WSS cluster geometrical-based stochastic model for UAV MIMO channels," IEEE T. Veh. Technol., vol. 71, no. 7, pp. 6884-6899, July, 2022, 10.1109/TVT.2022.3168193.

2) Section 3.3, which validates the monocular visual measurement method based on PPC distribution optimization, is sufficiently comprehensive. However, a concise summary of the data comparison results in this section is recommended to further strengthen its structural coherence.

Reply: Thank you for your review. A summary paragraph has been added to section 3.3 of the manuscript. The details are as follows:

From the above results, it can be seen that the monocular vision measurement method based on the optimisation of PPC allocation shows obvious advantages in different difficulty scenarios. Compared with DRM, Did-m3d, FAS, and SGM3D, the proposed method has higher AP values at all difficulty levels. The greatest improvement in detection performance was achieved in the “difficult” scenario. This indicates that the depth estimation and target detection models optimised for PPC allocation can provide higher accuracy and robustness when dealing with complex backgrounds, target occlusion and illumination changes. Furthermore, the proposed method effectively reduces false predictions and improves detection efficiency, underscoring its strong application potential in real-world monocular vision measurement tasks.

Compared with DRM, Did-m3d, FAS, and SGM3D, the proposed method has higher AP values...

In addition, compared with the traditional DRM, Did-m3d, FAS, and SGM3D, the PPC-optimised monocular vision measurement method...

3) The explanation for Figure 13 is overly brief, which may hinder readers’ intuitive understanding. It is suggested that the authors include a brief explanatory summary beneath the figure to improve clarity.

Reply: Thank you for your valuable suggestions. Additional explanations have been added to the corresponding text section of Figure 13. It is as follows:

The results of the study's suggested method for extracting PL points for each of the three data groups are compared with the points that is manually calibrated in Figure 13(a). The suggested method's extraction accuracy in experimental data group 1 is 95.30%. For experimental data groups 2 and 3, the extraction accuracy is 96.77% and 97.81%, respectively. The lower extraction accuracy in experimental data group 1 may be due to the fact that the topography of the region is more complex, with greater undulations and vertical shading. In contrast, the topography of the experimental area in data group 2 and data group 3 is relatively flatter, with less occlusion, higher PC density and less background interference. Therefore, the PCD of the groups 2 and 3 are more complete and the extraction accuracy is higher, which makes the PC extraction method more effective in identifying and extracting targets.

4) In the concluding section, the authors should incorporate specific references to figures or tables (e.g., “Figure 2 clearly demonstrates…” or “The data in Table 3 proves…”). Integrating such references with textual descriptions will enrich the paper’s structural integrity and analytical depth.

Reply: Thank you very much for your comments. Changes have been made to the conclusion section. Please see the revisions as follows:

Figure 9 reveals the superiority of the proposed method of the study in combination with PointNet++. Compared to F-PointNet+Transformer, the AP values of the proposed method in this study are improved by 1.35%, 1.50% and 1.33% at the three detection levels, respectively. Combined with the performance comparison test of different methods in Table 2, it can be seen that the average AP values of the proposed method of the study are improved by 16.41%, 14.76% and 7.85% compared to the other methods for different difficulty settings of IoUBEV=0.7, respectively. The above results show that by optimising the depth information of foreground targets, the accuracy of foreground targets in PPC can be improved, thus increasing the overall detection efficiency.

Overall Evaluation:

The aforementioned issues pertain to minor refinements in details and do not affect the core conclusions of the paper. If the authors can address these points, the manuscript is worthy of acceptance.

Reviewer #2:

The authors have addressed my previous comments. Thank you for that!

I have one minor suggestion: In Figure 8, the AP values for the unoptimized method appear to be increasing. Could the authors train the model until performance plateaus?

Reply: Thank you very much for your comments. The analysis of model convergence has been carried out by changing the number of model training iterations to 100. The details are as follows:

Meanwhile, the study utilizes the validation set of KITTI dataset for experiments. The encoder is trained on the training set of the KITTI data set for 100 epochs.

**Figure 8. Comparison of training results between two methods**

Figure 8 (a) shows the change curve of model training loss before and after optimization. It can be seen that as the number of iterations increases, the loss values of both models keep decreasing and gradually converge. When the number of iterations is about 60, the loss curve of the Transforme model converges. The Transformer optimised model proposed in this study does not show overfitting and its loss value is lower than the unoptimised model after 10 iterations.
